# Latissimus dorsi myocutaneous flap repair is effective after neoadjuvant chemotherapy for locally advanced breast cancer

**DOI:** 10.1186/s12957-022-02598-y

**Published:** 2022-04-27

**Authors:** Lu Li, Yue Yang, Wang Li, Xian Zhao, Jia He, Shuo Mei, Xuejun Guo, Xibin Zhang, Jianghua Ran

**Affiliations:** 1 Department of Breast Surgery, The Affiliated Calmette Hospital of Kunming Medical University, No. 1228, Beijing Road, Panlong District, Kunming, 650224 Yunnan China; 2 Department of Hepatopancreatobiliary Surgery, The Affiliated Calmette Hospital of Kunming Medical University, No. 1228, Beijing Road, Panlong District, Kunming, 650224 Yunnan China; 3 Department of Plastic Surgery, The Affiliated Calmette Hospital of Kunming Medical University, No. 1228, Beijing Road, Panlong District, Kunming, 650224 Yunnan China

**Keywords:** Breast cancer, Neoadjuvant chemotherapy, Latissimus dorsi musculocutaneous flap, Reconstruction, Modified radical mastectomy, Defect repair

## Abstract

**Objective:**

To describe the clinical outcome and physical condition of patients with locally advanced breast cancer (LABC) who received neoadjuvant chemotherapy followed by mastectomy and latissimus dorsi myocutaneous flap repair.

**Methods:**

A retrospective review of 142 patients with locally advanced breast cancer was selected from 1156 breast cancer patients in the South and North areas of The Affiliated Calmette Hospital of Kunming Medical University between May 2008 and December 2018.

**Results:**

All participants (*n* = 142) were women aged 40–55 years (average age 47.35 ± 0.43 years) who received neoadjuvant chemotherapy followed by mastectomy and latissimus dorsi flap repair. The median follow-up period was 16 months (range 12–24 months). For stage of disease, there were 19 cases (13%) in stage IIB, 31 cases (22%) in stage IIIA, 39 cases (28%) in stage IIIB, and 53 cases (37%) in stage IIIC, which were statistically significant with the physical condition of patients (≤ 0.001). Neoadjuvant chemotherapy was administered to shrink the tumors, and an average tumor size decrease from 10.05 ± 1.59 cm × (8.07 ± 1.54) cm to 6.11 ± 1.72 cm × (3.91 ± 1.52) cm (*P* < 0.001) was considered statistically significant. A t test was used for the ECOG score statistics, and the results showed that the scores were statistically significant (≤ 0.001) before and after neoadjuvant chemotherapy and after surgery.

**Conclusions:**

Neoadjuvant chemotherapy is an accepted treatment option for patients with locally advanced breast cancer, and the use of a latissimus dorsi musculocutaneous flap for post-mastectomy reconstruction may improve the patients’ physical condition. Our results indicated that this strategy was safe and feasible.

## Introduction

It is estimated that there were approximately 18.1 million new cancer cases worldwide in 2018, among which 2.1 million were breast cancer cases (11.6%). Breast cancer accounts for 24.2% of new cancer cases in 8.6 million women, ranking first among the cancers affecting women worldwide [1]. The incidence of breast cancer at all ages increases with income; however, the mortality rate from breast cancer is relatively high among women in the poorest countries [2]. In Yunnan Province, China, female breast cancer accounts for 14.60% of all malignant tumors, ranking first among female malignant tumors, and the mortality rate accounts for 6.48% of malignant tumors, ranking fifth among female malignant tumors. According to the multicenter breast disease survey in Yunnan, China, only 33.3% of patients had stage I, 49.2% of patients had stage II, 15.8% of patients had stage III, and 1.7% of patients had stage IV [3]. Thus, stage III and stage IV breast cancer patients still account for a large proportion. For the treatment of patients with LABC, there is still no common expert consensus, which requires clinicians to constantly explore the best treatment methods. LABC can be accompanied by ulceration and skin invasion, which seriously affects the patients’ quality of life (e.g., because of pain, unpleasant odors, and the need to frequently change the wound dressings) [4]. Unfortunately, this situation is difficult to manage effectively. Immediate breast reconstruction for T1–T2 tumors is generally considered safe and feasible [5]. However, the safety of immediate breast reconstruction for LABC remains the focus of much discussion [6]. Previous studies have demonstrated that chemotherapy and radiotherapy are preferred for patients with locally advanced breast cancer, and surgery is not the first choice [7].

At our center, we treated patients with locally advanced breast cancer using neoadjuvant chemotherapy followed by modified radical mastectomy and used latissimus dorsi musculocutaneous flaps to repair the resulting defects. Therefore, we aimed to examine the outcomes of this approach and share our experience. The objective of our study was to describe the clinical outcome and physical condition of patients with locally advanced breast cancer (LABC) who received neoadjuvant chemotherapy followed by mastectomy and latissimus dorsi myocutaneous flap repair.

## Material and methods

### Study sample

A total of 142 patients with locally advanced breast cancer were selected from 1156 breast cancer patients in the South and North areas of The Affiliated Calmette Hospital of Kunming Medical University between May 2008 and December 2018. All patients were assessed for treatment at the BCCA oncology assessment (including history, clinical examination, mammography, chest CT, laboratory examination, hepatobiliary, splenopancreatic ultrasound, and lung function) [8]. According to the AJCC classifications (8th edition), LABC is a category of breast cancer that includes T3, T4, any N2, or any N3 cases in TNM stage, from stage IIB to IIIC (including inflammatory breast cancer with no distant metastasis) [9]. Patients with inflammatory breast cancer, recurrence after breast-conserving surgery, and palliative care intentions were included. Some patients had pectoralis major muscle and skin invasion and ulceration and underwent neoadjuvant chemotherapy, and the maximum diameter of the tumor was reduced ≥ 30% (achieved a partial response, PR) before undergoing modified radical mastectomy [10]. Immediately after resection, a latissimus dorsi musculocutaneous flap was applied to the related defect. This retrospective study evaluated the patients’ outcomes and physical condition.

### Ethics statement

This research was conducted ethically in accordance with the tenets of the Declaration of Helsinki. The study protocol was approved by the Medical Ethics Committee of The Affiliated Calmette Hospital of Kunming Medical University (Document Number: 2019-02). All patients were delabelled.

### Chemotherapy

All patients received hollow needle biopsy before neoadjuvant chemotherapy and were pathologically confirmed as malignant breast tumors. Based on the National Comprehensive Cancer Network guidelines [4], all patients underwent 4–6 cycles of neoadjuvant treatment with anthracycline combined with taxane chemotherapy, including TEC, EC-T, or TAC (cyclophosphamide, 600 mg/m^2^ intravenously on day 1, epirubicin 70 mg/m^2^ intravenously on day 1, and Taxol 90 mg/m^2^ intravenously on day 2); each interval lasted for 21 days. All patients completed six and four preoperative cycles, respectively. Among them, 48 (33.8%) received TEC chemotherapy, 86 (60.6%) received EC-T, and 8 (5.6%) received TAC. After the end of neoadjuvant chemotherapy, the maximum tumor diameter of all patients was reduced ≥ 30% (achieved a partial response, PR) [10]. All patients received corresponding adjuvant treatment (endocrine therapy, trastuzumab treatment) after chemotherapy and surgery.

### Preoperative evaluations

Ulcer secretions were submitted for bacterial culture, and anti-inflammatory treatment was provided for 3–5 days when local inflammation was obvious. Blood flow in the ipsilateral thoracic dorsal artery was examined using a Doppler flow detector (SIEMENS ACUSON X150), which revealed normal blood flow for all patients. After 4–6 cycles of chemotherapy, the tumor sizes decreased, bleeding decreased, and the wounds exhibited cleaner surfaces. Physical condition was assessed according to the status score proposed by the Eastern Oncology Cooperative Group [11], with a score of ≤ 2 indicating that surgery could be tolerated. The patients also exhibited improvements in their general condition, which made them able to tolerate surgical treatment.

### Surgical treatment

**Fig. 1:**
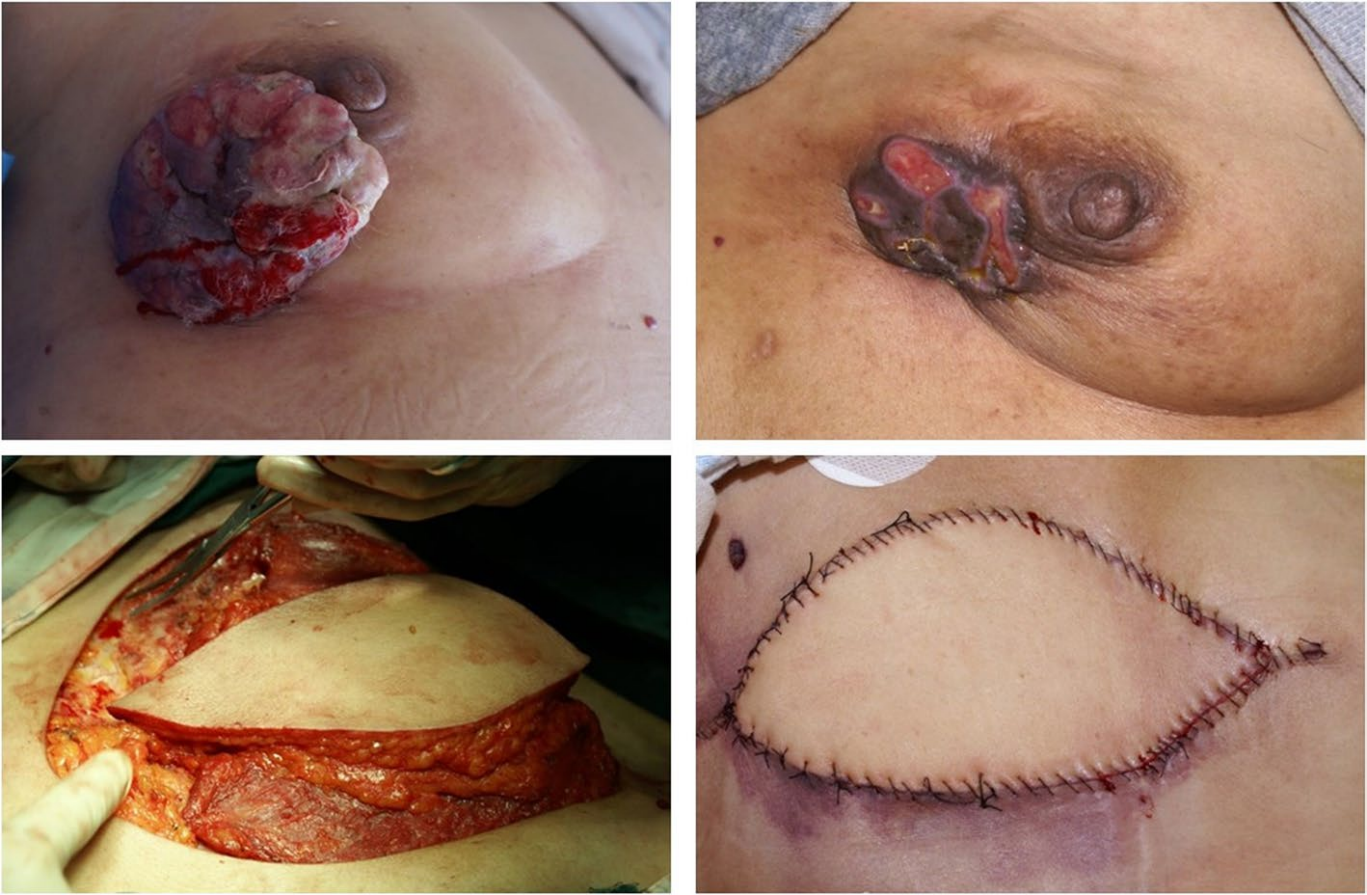
Reconstruction of latissimus dorsi myocutaneous flap after neoadjuvant chemotherapy for locally advanced breast cancer

The first step involved resection with a 1–2-cm margin around the ulcers and any surrounding skin lesions. The flap was separated to preserve the subdermal vascular network and adipose tissue. High-frequency electrotomy was used to lift and separate the breast tissue from the medial sternal edge, and the glandular tissue was removed with the pectoralis major fascia below the tumor and the pectoralis major invaded by the tumor (the pectoralis major was left uninvaded) from the top down (parallel to the pectoralis major fibers). The ipsilateral axillary lymph nodes were dissected, similar to the modified radical mastectomy. Keep the long thoracic nerve, thoracic dorsal nerve, and subscapular vessels. The excised tissue was immediately sent to the pathology laboratory for a rapid examination of the frozen section. After confirming that the margins were free of tumor cells, the defect size and shape were considered to design and elevate an ipsilateral latissimus dorsi musculocutaneous flap, which was then transferred to the defect. The flap was designed to be 1–2 cm longer and wider than the defect, with the pedicle length determined based on the distance between the wound surface and the latissimus dorsi musculocutaneous flap. Patients were changed from supine position to lateral position for secondary disinfection. The latissimus dorsi flap was opened from the defect area to the axilla. The skin and superficial fascia were cut, and the deep adipose tissue beneath the fascia was lifted together with the latissimus dorsi to form a large tissue, and the fibrous connection between the serratus anterior and the latissimus dorsi was cut. Protect the vascular nerve pedicle. The flap was transferred to the wound’s surface while avoiding torsion on the pedicle during the transfer and excessive tension during suturing [12]. The third step involved the primary closure of the donor-site defect, although larger defects were repaired using a medium-thickness skin graft. Vacuum drainage tubes were placed in the chest wall and the ipsilateral axilla, with daily monitoring of the drainage volume. When the drainage flow is less than 20 ml drainage tube (Fig. 1), the flap’s blood supply was monitored daily, and the dressings were changed every other day. Antibiotic treatment was provided where necessary based on the results from the preoperative wound bacterial cultures. A total of 137 patients underwent post-operative radiotherapy; ER- and PR-positive patients received endocrine therapy, and 47 HER-2-positive patients received trastuzumab treatment.

### Statistical analysis

To analyze control variables, the relationships between NACT, surgery, and physical condition were compared using multivariate variance analysis, and continuous variables were evaluated using the *T* test. According to the literature [13, 14], we analyzed known confounding factors, including age, BMI, smoking history, menopausal status, etc. In addition, stage and grade were investigated based on clinical experience, and this analysis allows for a more accurate comparison. Statistical analysis was performed using SPSS 25.0 for Windows statistics program (IBM Corp, Armonk, NY), and *P* values less than 0.05 indicated significant differences.

## Results

A total of 142 patients with locally advanced breast cancer were selected from 1156 breast cancer patients between May 2008 and December 2018. The patient demographic and tumor characteristics of the patients are shown in Table 1. All participants (*n* = 142) were women aged 40–55 years (average age 47.35 ± 0.43 years). The median follow-up period was 16 months (range 12–24 months). Among the pathological types of all patients, 138 cases (97%) were infiltrating ductal carcinoma, and 4 cases (3%) were inflammatory. The molecular subtyping in these 142 cases was the luminal A (ER+, PR+, HER2−) subtype in 38 cases (26.8%), the luminal B (ER+, PR+, HER2+) subtype in 52 cases (36.6%), HER-2 overexpression in 21 cases (14.8%), and triple negative in 31 cases (21.8%). There were 106 cases (75%) involving skin and 53 cases (37%) involving pectoralis major. Related confounders, including age, BMI, smoking history, and menopausal status, were not statistically significant in this study (≥ 0.05). Since the grades of locally advanced breast cancer are all T3 and T4 cases, these cases were selected in this study. There were 36 (25%) cases with tumor grade T3 and 106 (75%) cases with tumor grade T4, which was statistically significant with physical condition after neoadjuvant chemotherapy and surgery (≤ 0.001). Regarding the stage of the disease, there were 19 cases (13%) in stage IIB, 31 cases (22%) in stage IIIA, 39 cases (28%) in stage IIIB, and 53 cases (37%) in stage IIIC, which were statistically significant with the physical condition of patients (≤ 0.001).

**Table 1:** Patient and tumor characteristics

Variable	Value	*P* value
Age (year), median (range)	47.35 ± 3.97 (40–55)	≥ 0.05
Smoking, *n* (%)		≥ 0.05
Yes	7(5%)	
No	135(95%)	
BMI, median (range)	25.03 ± 2.1(20–32)	≥ 0.05
Follow-up (months), median (range)	16.5 ± 3.73(12–24)	≥ 0.05
Menopausal status, *n* (%)		≥ 0.05
Premenopausal	47(33%)	
Post-menopausal	95(67%)	
Stage of disease, *n* (%)		≤ 0.05
IIB	19(13%)	
IIIA	31(22%)	
IIIB	39(28%)	
IIIC	53(37%)	
Tumor classification, *n* (%)		≤ 0.001
T1	0	
T2	0	
T3	36(25%)	
T4	106(75%)	
Lymph node status, *n* (%)		≤ 0.05
N0	0	
N1	48(34%)	
N2/N3	94(66%)	
ER, PR status at initial diagnosis, *n* (%)		≥ 0.05
Positive	90(63.4%)	
Negative	52(33.6%)	
HER-2 status at initial diagnosis		≥ 0.05
Positive	41(28.9%)	
Negative	101(71.1%)	
Surgical margin status, *n* (%)		≥ 0.05
Positive	2(1%)	
Negative	140(99%)	
Skin invasion and ulceration, *n* (%)		
Yes	106(75%)	
No	36(25%)	
Pectoralis major		
Yes	53(37%)	
No	89(63%)	
Histological type, *n* (%)		
Infiltrating ductal carcinoma	138(97%)	
Inflammatory	4(3%)	
Molecular subtyping, *n* (%)		≤ 0.05
Luminal A	38(26.8%)	
Luminal B	52(36.6%)	
Triplenegative	31(21.8%)	
HER-2 overexpression	21(14.8%)	

**Fig. 2:**
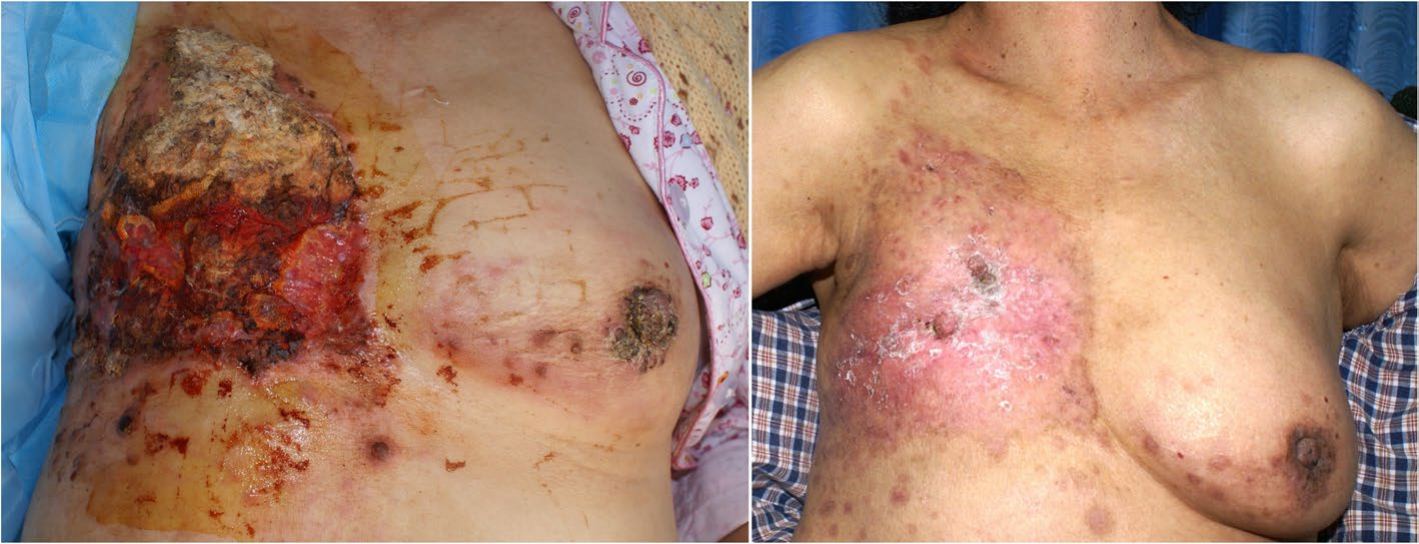
Neoadjuvant chemotherapy for locally advanced breast cancer

All participants had an average tumor size of (10.05 ± 1.59) cm × (8.07 ± 1.54) cm (maximum 15.3 cm × 12.6 cm, minimum 8.0 cm × 6.5 cm). All patients underwent 4–6 cycles of neoadjuvant treatment with anthracycline combined with taxane chemotherapy, including TEC, EC-T, or TAC, each interval lasted for 21 days. All patients completed six and four preoperative cycles, respectively, achieved a partial response. Therefore, 4–6 courses of neoadjuvant chemotherapy were administered to shrink the tumors, bleeding decreased and an average tumor size decrease from 10.05 ± 1.59 cm × (8.07 ± 1.54) cm to 6.11 ± 1.72 cm × (3.91 ± 1.52) cm (*P* < 0.001) was considered statistically significant (Table 2). Physical condition ECOG score of ≤ 2 was indicating that surgery could be tolerated. All 137 patients underwent modified radical mastectomy and latissimus dorsi musculocutaneous flap transfer to repair the defect (Table 2). There were 2 cases (1.46%) had partial necrosis in the suture area at the edge of the flap. It was considered that excessive suture tension caused daily dressing change and scar healing three weeks later. And 8 cases (5.84%) of seroma at donor site (Table 3). Negative pressure attraction was maintained and dressing was changed daily, which healed spontaneously 2 weeks later. Endocrine therapy was given to 90 ER- and PR-positive patients (63.4%). Twenty-eight patients with positive HER-2 received trastuzumab treatment voluntarily (19.7%).The median follow-up period was 16 months (range 12–24 months), with 3 patients developing pulmonary metastasis at 14 months after surgery. None of the patients who received chemotherapy and surgery (*n* = 137) died during a follow-up period of 12 to 24 months (median follow-up period was 16 months). However, 5 patients who received neoadjuvant chemotherapy refused surgery and died during the follow-up period (Table 4) (Fig. 2).

**Table 2:** Tumor size comparison $\left(\overline{X}\pm SD\right)$

	Case (*n*)	Average tumor size (cm × cm)
Prechemotherapy	142	(10.05 ± 1.59) cm × (8.07 ± 1.54) cm
Post-chemotherapy	142	(6.11 ± 1.72) cm × (3.91 ± 1.52) cm
Post-operation		0
*P*		< 0.001

**Table 3:** Complications

		Surgery after NAC *N* = 137	NAC without surgery *N* = 5	*p* value
Complications	Partial flap necrosis	2(1.46%)	–	
*n*, (%)	Infection	0	–	
	Seroma	8(5.84%)	–	
	Bleeding	0	–	
	Total	10(7.3%)	5(3.5%)	≤ 0.001
Recurrences	Local recurrences	0	5(3.5%)	≤ 0.001
*n*, (%)	Metastasis	3(2.19%)	5(3.5%)	≤ 0.001
Dead *n*, (%)			5(3.5%)	≤ 0.001

*NAC* neoadjuvant chemotherapy

**Table 4:** Treatment characteristics

Treatment, *n* = 142	Value	*P* value
NACT, *n* (%)	142	≤ 0.001
TEC	48 (33.8%)	
EC-T	86 (60.6%)	
TAC	8 (5.6%)	
Endocrine therapy, *n* (%)		≥ 0.05
Yes	90 (63.4%)	
No	52 (36.6%)	
Trastuzumab treatment, *n* (%)		≥ 0.05
Yes	28 (19.7%)	
No	114 (80.3%)	
Surgical, *n* (%)	137 (96.5%)	≤ 0.001
Rejection surgery, *n*	5	
Death, *n*	5	≤ 0.001
Survival, *n*	137

The physical condition of the patients was scored according to the Eastern Oncology Cooperative Group (ECOG) (Tables 5 and 6) [11]. The scores of these patients were all 2–3 points; the *T* test was used for statistics, and the results showed that the scores were statistically significant (*P* ≤ 0.001) before and after neoadjuvant chemotherapy and after surgery. This indicates that neoadjuvant chemotherapy and surgery have significantly improved the physical condition of patients. This is especially true in patients with skin invasion and ulceration.

**Table 5:** Physical condition (ECOG)

	Prechemotherapy	Post-chemotherapy	Post-operation	Non-operating	*P* value
	*n* = 137			*n* = 5	
II B	1.5 ± 0.45	1 ± 0.32	0.48 ± 0.37		≤ 0.001
III A	1.77 ± 0.26	1.36 ± 0.32	0.91 ± 0.44		≤ 0.001
III B	2.36 ± 0.67	1.59 ± 0.44	1 ± 0.32		≤ 0.001
III C	2.78 ± 0.34	1.74 ± 0.52	1.32 ± 0.34		≤ 0.001
Skin invasion and ulceration					
Yes	3.26 ± 0.57	2.14 ± 0.25	1.43 ± 0.42		≤ 0.001
No	2.51 ± 0.48	1.76 ± 0.31	1.21 ± 0.24		≤ 0.001

**Table 6:** Core tip

Treatment		Procedure	Aim
Chemotherapy	Four to six course of treatment	TEC	The tumor wound was reduced and blood supply was reduced. ECOG score ≤ 3
		EC-T	
		TAC	
Surgery	Preoperative	The blood supply of the flap was investigated by ultrasound Doppler. Remove ulcer secretions for bacterial culture. According to the results of bacterial culture, sensitive antibiotics were selected.	Excise tumor, repair defect and improve patient's physical condition..
	Intraoperative	The excised tissue was sent for pathological and cryogenic examination. Try to achieve negative margin. The latissimus dorsi flap was designed according to the scope of resection. Donor site suture, cannot suture skin graft.	
	Post-operation	Antibiotics were given according to the results of preoperative bacterial culture. Continuous negative pressure drainage, drainage liquid less than 20 ml, remove the drainage tube.	
Radiotherapy		Patients with lymph node metastasis were treated with radiotherapy in stage III and IV.	Reduce local tumor recurrence, reduce distant metastasis.
Endocrinotherapy		ER, PR-positive patients	
Trastuzumab treatment		Patients with positive HER-2	

## Discussion

At present, neoadjuvant chemotherapy has become the standard treatment mode for patients with locally advanced breast cancer. Studies of NSABP-B18 and B-27 [15, 16] showed that neoadjuvant chemotherapy can increase the chance of breast-conserving surgery for breast cancer, improve the quality of life of patients, improve disease-free survival and overall survival of patients, and improve the prognosis of patients. Anthracycline and taxane chemotherapeutic agents have long been the basis of adjuvant chemotherapy for breast cancer patients and are equally important in preoperative chemotherapy. The study [17] showed that the TEC regimen was superior to the FEC regimen in terms of the overall response rate and PCR in neoadjuvant chemotherapy, with statistically significant differences. Therefore, the TEC (TAC) regimen was selected for preoperative neoadjuvant chemotherapy in our center.

Neoadjuvant chemotherapy is also widely accepted for managing locally advanced breast cancer, which often allows the patient to undergo subsequent surgical treatment. For example, neoadjuvant chemotherapy using doxorubicin and cyclophosphamide allowed surgery in 84% of patients with locally advanced breast cancer that was initially considered inoperable [18]. In this setting, neoadjuvant chemotherapy typically involves 4–6 cycles, as additional cycles do not generally increase the therapeutic effect [19]. Therefore, in our study, we chose 4–6 cycles of chemotherapy. According to Choi J, breast pathology complete response (PCR) was achieved in 105 patients (27.5%) [20]. This is consistent with our research, 4–6 courses of neoadjuvant chemotherapy can shrink the tumor (*P* < 0.001), reduce bleeding. Furthermore, neoadjuvant chemotherapy has the same efficacy as conventional adjuvant chemotherapy and helps to downstage the tumor to increase the possibility of less extensive breast-conserving surgery and the successful implementation of other operations [20]. These effects were observed in our patients, who experienced reductions in their wound area and mass size, with downstaging to the point that radical resection was feasible. There are many regimens for neoadjuvant chemotherapy, although the TEC regimen is considered classic because it does not have an elevated risk of adverse reactions and does not significantly affect wound healing [21]. The toxic effects of the TEC regimen mainly involve leukopenia, hair loss, nausea, and vomiting, which can be managed using symptomatic treatment. These characteristics suggest that the TEC regimen is safe and feasible in this setting.

According to previous studies [6], the safety of immediate surgery for T4 tumors remains to be proven. Based on previous studies [22], stage T4 tumors of breast cancer, especially those associated with skin and pectoralis major invasion, are considered unsuitable for immediate surgery and are often excluded from breast-conserving surgery. However, chemotherapy or radiotherapy alone cannot completely relieve the patients’ pain and local ulceration or improve their quality of life. Based on our previous observations and studies, chemotherapy alone can reduce the tumor size and improve the physical condition of patients, and the ECOG score of the patient’s physical condition is ≤ 2 (*P* ≤ 0.001), which can tolerate surgery.. However, a tumor cannot be completely eliminated by chemotherapy, patients still do not achieve tumor-free survival, and the quality of life still cannot be significantly improved.

Locally advanced breast cancer does not have a clear definition, although it generally involves a primary tumor with a diameter of > 5cm (T3), any size of tumor that has spread to the chest wall or skin (T4), or palpable axillary lymph nodes (stage III) [23]. Patients with locally advanced breast cancer often experience pain, ulceration, bleeding, and other adverse effects, which contribute to their physiological and psychological burden. The physical condition of patients was scored according to the Eastern Oncology Cooperative Group [11]. The scores of these patients were all 2–3 or 3–4 points.

All patients in this study had LABC with T3 and T4 tumors, some accompanied by skin invasion or ulcers. Immediate repair is difficult in such cases due to intraoperative trauma and inability to suture the skin after excision. However, patients at our center may undergo neoadjuvant chemotherapy, which shrinks the tumor and the area of ulceration and, thus, a less traumatic mastectomy and a possible immediate repair. We believe that this approach benefits patients, as it provides the greatest reduction in tumor burden and improves their quality of physical condition (*P* ≤ 0.001), although it requires good coordination between the surgeons performing the modified mastectomy and reconstruction. This technique also agrees with the current practice of cancer-related plastic surgery [24], in which the effects of any adjuvant chemotherapy should be considered when determining whether the patient is eligible for reconstructive surgery. Therefore, we assessed the patient's quality of life and tumor size before immediate plastic surgery and concluded that the patient could tolerate surgery and that the transferred latissimus dorsi flap could repair the wound defect. Reconstruction is generally performed after the patient has achieved a treatment response, although the technique and timing must be tailored to the individual patient.

A large skin incision is needed to achieve a negative resection margin when treating locally advanced breast cancer, especially in cases of stage IIIB or IIIC disease [7]. Thus, wound coverage has become a key issue, as it is important to provide coverage of extensive chest wall defects. Unfortunately, a large incision is needed to achieve a sufficient negative resection margin, and post-operative wound coverage has become a key issue [25]. Conventional skin grafting significantly affects the post-operative appearance and morphology, and these grafts have a poor tolerance of post-operative radiotherapy and a risk of serious radiation ulcers [26]. However, flap-based reconstruction is safe for post-operative radiotherapy and is often used to manage radiation ulcers. Masaki et al. reported a successful case of a large abdominal wall defect treated by a combined serratus anterior and latissimus dorsi musculocutaneous flap [27]. Therefore, an autologous musculocutaneous flap would be ideal for reconstruction immediately after breast cancer surgery [28]. Latissimus dorsi musculocutaneous flaps can be combined with local flaps as a “combined pedicled flap” to repair larger defects in women with large breasts or breast ptosis [29]. Furthermore, the latissimus dorsi musculocutaneous flap is mainly supplied by the subscapular and thoracodorsal arteries, which have not been damaged during radical mastectomy with axillary lymph node dissection [30]. Moreover, that operation is relatively simple, has minimal effects on the donor region at the shoulder and the back, and has few serious complications [31]. Long-term results of immediate replacement of volume with latissimus dorsi musculocutaneous flaps for breast conserving yielded satisfactory and stable aesthetic results that remained unchanged over a long period of time. This option should be considered when indications for post-mastectomy radiotherapy exist or may exist [32]. Skin-sparing mastectomy (SSM) and immediate breast reconstruction (IBR) with latissimus dorsi flaps are safe and produce high patient satisfaction [33]. Some surgeons are hesitant to perform immediate breast reconstruction on high-risk patients. According to Dudley CM. et al. [34], a study of 5318 patients with stages II and III breast cancer who underwent immediate breast reconstruction compared with unilateral mastectomy showed similar rates of ipsilateral local recurrence. This provides the surgeon with an acceptable option. Other studies have shown that IBR is a safe option for patients with locally advanced breast cancer without adverse effects on survival, cancer recurrence, or adjuvant therapy [35].

In our cases, rapid pathological examination was performed to confirm the negative surgical margins. The results indicated that skin grafting alone would not be an appropriate option, especially given the limited tolerance of these flaps to radiotherapy [26]. The rectus abdominis flap is also fairly close to the related defect, although damage to the blood supply during removal of the anterior rectus sheath and muscle might compromise its use in this setting [36]. Furthermore, women may have undergone previous abdominal procedures (e.g., liposuction or plastic surgery), which would exclude the use of transverse rectus abdominis myocutaneous and deep inferior epigastric perforator flaps for breast reconstruction [37]. Therefore, the latissimus dorsi musculocutaneous flap is an ideal choice for repairing the defect left by radical mastectomy [38].

Radical mastectomy is ideal when there is no distant metastasis in cases of locally advanced breast cancer, although surgery should still aim to minimize the tumor burden even in cases where radical resection is not possible. In these cases, surgery helps prolong the patients’ lives and improves their quality of life [39]. Latissimus dorsi musculocutaneous flaps have advantages in this case. According to an analysis of short-term and long-term outcomes of breast reconstruction surgery immediately following neoadjuvant chemotherapy in 1135 patients with IBR and NAC, the valuation of complications showed that there were no cases of total flap necrosis, 2 cases of partial flap necrosis, 1 case of wound infection, and only 1 case requiring delay of follow-up treatment due to partial flap necrosis. A long-term assessment showed no local recurrence, but distant metastasis was observed in 4 cases, and 3 patients died [40]. This statistical result is basically consistent with our research results. Therefore, we believe that our study is safe and effective and can improve the quality of life of patients.

Our study had some limitations. Specifically, its sample size was small, and it was a short-term study without a control group. Further prospective studies are needed. Long-term observation and analysis are required to assess overall survival.

## Conclusions

In conclusion (Table 6), neoadjuvant chemotherapy is an accepted treatment option for patients with locally advanced breast cancer, and the use of a latissimus dorsi musculocutaneous flap for post-mastectomy reconstruction may improve the patients’ physical condition. Our results indicated that this strategy was safe and feasible due to the lack of significant donor-site and reconstruction-related complications. However, long-term follow-up is needed to confirm the safety and efficacy of this strategy.

## Data Availability

The datasets used and/or analyzed during the current study are available from the corresponding author on reasonable request.

## References

[1] Bray F, Ferlay J, Soerjomataram I, Siegel RL, Torre LA, Jemal A (2015). Global cancer statistics 2018: GLOBOCAN estimates of incidence and mortality worldwide for 36 cancers in 185 countries. CA Cancer J Clin.

[2] Bellanger M, Zeinomar N, Tehranifar P, Terry MB (2018). Are global breast cancer incidence and mortality patterns related to country-specific economic development and prevention strategies?. J Glob Oncol.

[3] Li Y, Xi Y, Liu Q, Zhang N, Nie J (2014). Baseline characteristics of breast cancer in Yunnan Province, China. Mod Onco.

[4] NCCN Clinical Practice Guidelines Oncology. Breast Cancer. 2019. https://www.nccn.org/professionals/physician_gls/default.aspx

[5] Bertozzi N, Pesce M, Santi P, Raposio E (2017). One-stage immediate breast reconstruction: a concise review. Biomed Res Int.

[6] Wang M, Chen H, Wu K, Ding A, Zhang P, Zhang M (2019). Post-mastectomy immediate breast reconstruction is oncologically safe in well-selected T4 locally advanced breast cancer: a large population-based study and matched case-control analysis. Breast Cancer Res Treat.

[7] Koppiker CB, Noor AU, Dixit S, Busheri L, Sharan G, Dhar U, Allampati HK, Nare S (2019). Extreme oncoplastic surgery for multifocal/multicentric and locally advanced breast cancer. Int J Breast Cancer.

[8] Ho AL, Tyldesley S, Macadam SA, Lennox PA (2012). Skin-sparing mastectomy and immediate autologous breast reconstruction in locally advanced breast cancer patients: a UBC perspective. Ann Surg Oncol.

[9] Pellicciaro M, Materazzo M, Buonomo C, Vanni G (2021). Feasibility and oncological safety of axillary reverse mapping in patients with locally advanced breast cancer and partial response after neoadjuvant chemotherapy. In Vivo.

[10] Cortazar P, Geyer CE (2015). Pathological complete response in neoadjuvant treatment of breast cancer. Ann Surg Oncol.

[11] Wildes TM, Ruwe AP, Fournier C, Gao F, Carson KR, Piccirillo JF, Tan B, Colditz GA (2013). Geriatric assessment is associated with completion of chemotherapy, toxicity, and survival in older adults with cancer. J Geriatr Oncol.

[12] Motono N, Shimada K, Kamata T, Uramoto H (2019). Sternal resection and reconstruction for metastasis due to breast cancer: the Marlex sandwich technique and implantation of a pedicled latissimus dorsi musculocutaneous flap. J Cardiothorac Surg.

[13] Yang JR, Kuo WL, Yu CC, Chen SC, Huang JJ (2021). Reconstructive outcome analysis of the impact of neoadjuvant chemotherapy on immediate breast reconstruction: a retrospective cross-sectional study. BMC Cancer.

[14] Wengler CA, Valente SA, Al-Hilli Z, Woody NM, Muntean JH, Abraham J (2017). Determinants of short and long term outcomes in patients undergoing immediate breast reconstruction following neoadjuvant chemotherapy. J Surg Oncol.

[15] Wolmark N, Wang J, Mamounas E, Bryant J, Fisher B (2001). Preoperative chemotherapy in patients with operable breast cancer: nine-year results from National Surgical Adjuvant Breast and Bowel Project B-18. J Natl Cancer Inst Monogr.

[16] Bear HD, Anderson S, Smith RE, Geyer CE, Mamounas EP, Fisher B (2006). Sequential preoperative or postoperative docetaxel added to preoperative doxorubicin plus cyclophosphamide for operable breast cancer: National Surgical Adjuvant Breast and Bowel Project Protocol B-27. J Clin Oncol.

[17] Wang B, Fu JF, Hong ZW (2009). Efficacy of neoadjuvant chemotherapy with FEC and TEC regimen on breast cancer. Ai Zheng.

[18] Wu J, Cao G, Sun X, Lee J, Rubin DL, Napel S, Kurian AW, Daniel BL, Li R (2018). Intratumoral spatial heterogeneity at perfusion MR imaging predicts recurrence-free survival in locally advanced breast cancer treated with neoadjuvant chemotherapy. Radiology..

[19] Brackstone M, Palma D, Tuck AB, Scott L, Potvin K, Vandenberg T, Perera F, D'Souza D, Taves D, Kornecki A, Muscedere G, Chambers AF (2017). Concurrent neoadjuvant chemotherapy and radiation therapy in locally advanced breast cancer. Int J Radiat Oncol Biol Phys.

[20] Choi J, Laws A, Hu J, Barry W, Golshan M, King T (2018). Margins in breast-conserving surgery after neoadjuvant therapy. Ann Surg Oncol.

[21] Liu Y, Xu Z, Zhang Z, Wen G, Sun J, Han F (2019). Efficacy and safety of TE/TEC/intensive paclitaxel neoadjuvant chemotherapy for the treatment of breast cancer. Oncol Lett.

[22] Mazor AM, Mateo AM, Demora L, Sigurdson ER, Handorf E, Daly JM, Aggon AA, Anderson PR, Weiss SE, Bleicher RJ (2019). Breast conservation versus mastectomy in patients with T3 breast cancers (> 5 cm): an analysis of 37,268 patients from the National Cancer Database. Breast Cancer Res Treat.

[23] Priyadarshini R, Raj GM, Kayal S, Ramesh A, Shewade DG (2019). Influence of ABCB1 C3435T and C1236T gene polymorphisms on tumour response to docetaxel-based neo-adjuvant chemotherapy in locally advanced breast cancer patients of South India. J Clin Pharm Ther.

[24] Scomacao I, AlHilli Z, Schwarz G (2020). The Role of Oncoplastic Surgery for Breast Cancer. Curr Treat Options Oncol.

[25] Tanos G, Prousskaia E, Chow W, Angelaki A, Cirwan C, Hamed H, Farhadi J (2016). Locally advanced breast cancer: autologous versus implant-based reconstruction. Plast Reconstr Surg Glob Open.

[26] Khan AA, Khan IM, Nguyen PP, Lo E, Chahadeh H, Cerniglia M, Noriega JA (2020). Skin Graft Techniques. Clin Podiatr Med Surg.

[27] Fujioka M, Hayashida K, Morooka S, Saijo H, Nonaka T (2014). Combined serratus anterior and latissimus dorsi myocutaneous flap for obliteration of an irradiated pelvic exenteration defect and simultaneous site for colostomy revision. World J Surg Oncol.

[28] Cagli B, Manzo MJ, Tenna S, Piombino L, Poccia I, Persichetti P (2012). Heterologous reconstruction and radiotherapy: the role of latissimus dorsi flap as a salvage. Acta Chir Plast.

[29] Lee S, Lee J, Lee S, Bae Y (2014). Oncoplastic breast surgery with latissimus dorsi myocutaneous flap for large defect in patients with ptotic breasts: is it feasible when combined with local flaps?. World J Surg Oncol.

[30] Aomatsu N, Tei S, Haraoka G, Hosoi K, Fujii N, Tsujio G, Hiramatsu S, Wang E, Iwauchi T, Morimoto J, Nishii T, Kosaka K, Uchima Y, Takeuchi K (2015). A case of locally advanced breast cancer treated with modified radical mastectomy with immediate reconstruction using a tissue expander after endocrine therapy. Gan To Kagaku Ryoho.

[31] Chen H, Zhang P, Zhang M, Wang M, Bai F, Wu K (2019). Growing trends of contralateral prophylactic mastectomy and reconstruction in young breast cancer. J Surg Res.

[32] Hernanz F, Sánchez S, Cerdeira MP, Figuero CR (2011). Long-term results of breast conservation and immediate volume replacement with myocutaneous latissimus dorsi flap. World J Surg Oncol.

[33] Kim Z, Kang SG, Roh JH, Park JH, Lee J, Kim S, Lim CW, Lee MH (2012). Skin-sparing mastectomy and immediate latissimus dorsi flap reconstruction: a retrospective analysis of the surgical and patient-reported outcomes. World J Surg Oncol.

[34] Dudley CM, Wiener AA, Stankowski-Drengler TJ, Schumacher JR, Francescatti AB, Poore SO, Greenberg CC, Neuman HB (2021). Rates of ipsilateral local-regional recurrence in high-risk patients undergoing immediate post-mastectomy reconstruction (AFT-01). Clin Breast Cancer.

[35] Taqi K, Pao JS, Chen L, Ma C, Zhang M, McKevitt E, Bazzarelli A, Dingee C, Warburton R (2021). Immediate breast reconstruction in locally advanced breast cancer: is it safe?. Breast Cancer Res Treat.

[36] Lee SB, Lee JW, Kim HJ, Ko BS, Son BH, Eom JS, Lee TJ, Ahn SH (2018). Long-term outcomes of patients with breast cancer after nipple-sparing mastectomy/skin-sparing mastectomy followed by immediate transverse rectus abdominis musculocutaneous flap reconstruction: Comparison with conventional mastectomy in a single center study. Medicine (Baltimore).

[37] Zoghbi Y, Gerth DJ, Tashiro J, Golpanian S, Thaller SR (2017). Deep inferior epigastric perforator versus free transverse rectus abdominis myocutaneous flap: complications and resource utilization. Ann Plast Surg.

[38] Rezaei E, Pouryousef K, Karimi M, Hajebi Khaniki S, Baradaran SE (2019). Latissimus dorsi musculocutaneous flap inset innovation in breast reconstruction. World J Plast Surg.

[39] Youssef MMG, Namour A, Youssef OZ, Morsi A (2018). Oncologic and cosmetic outcomes of oncoplastic breast surgery in locally advanced breast cancer after neoadjuvant chemotherapy, experience from a developing country. Indian J Surg Oncol.

[40] Ishiba T, Aruga T, Miyamoto H, Ishihara S, Nara M, Adachi M, et al. Short- and long- term outcomes of immediate breast reconstruction surgery after neoadjuvant chemotherapy. Surg Today. 2021. 10.1007/s00595-021-02316-3. 10.1007/s00595-021-02316-3 34089365

